# A provably lightweight and secure DSSE scheme, with a constant storage cost for a smart device client

**DOI:** 10.1371/journal.pone.0301277

**Published:** 2024-04-25

**Authors:** Salim Sabah Bulbul, Zaid Ameen Abduljabbar, Rana Jassim Mohammed, Mustafa A. Al Sibahee, Junchao Ma, Vincent Omollo Nyangaresi, Iman Qays Abduljaleel

**Affiliations:** 1 Directorate General of Education Basra, Ministry of Education, Basra, Iraq; 2 Department of Computer Science, College of Education for Pure Sciences, University of Basrah, Basrah, Iraq; 3 National Engineering Laboratory for Big Data System Computing Technology, Shenzhen University, Shenzhen, PR China; 4 Computer Technology Engineering Department, Iraq University College, Basrah, Iraq; 5 College of Big Data and Internet, Shenzhen Technology University, Shenzhen, China; 6 Department of Computer Science and Software Engineering, Jaramogi Oginga Odinga University of Science & Technology, Bondo, Kenya; 7 Department of Applied Electronics, Saveetha School of Engineering, SIMATS, Chennai, Tamil Nadu, India; 8 Department of Computer Science, College of Computer Science and Information Technology, University of Basrah, Basrah, Iraq; Sunway University, MALAYSIA

## Abstract

Outsourcing data to remote cloud providers is becoming increasingly popular amongst organizations and individuals. A semi-trusted server uses Searchable Symmetric Encryption (SSE) to keep the search information under acceptable leakage levels whilst searching an encrypted database. A dynamic SSE (DSSE) scheme enables the adding and removing of documents by performing update queries, where some information is leaked to the server each time a record is added or removed. The complexity of structures and cryptographic primitives in most existing DSSE schemes makes them inefficient, in terms of storage, and query requests generate overhead costs on the Smart Device Client (SDC) side. Achieving constant storage cost for SDCs enhances the viability, efficiency, and easy user experience of smart devices, promoting their widespread adoption in various applications while upholding robust privacy and security standards. DSSE schemes must address two important privacy requirements: forward and backward privacy. Due to the increasing number of keywords, the cost of storage on the client side is also increasing at a linear rate. This article introduces an innovative, secure, and lightweight Dynamic Searchable Symmetric Encryption (DSSE) scheme, ensuring Type-II backward and forward privacy without incurring ongoing storage costs and high-cost query generation for the SDC. The proposed scheme, based on an inverted index structure, merges the hash table with linked nodes, linking encrypted keywords in all hash tables. Achieving a one-time O(1) storage cost without keyword counters on the SDC side, the scheme enhances security by generating a fresh key for each update. Experimental results show low-cost query generation on the SDC side (6,460 nanoseconds), making it compatible with resource-limited devices. The scheme outperforms existing ones, reducing server-side search costs significantly.

## 1 Introduction

With the emergence of big data and its applications [1–5], cloud computing is becoming increasingly important and popular for the Smart Device Client (SDC). Since sensitive user information can be easily hacked, such information is encrypted on the SDC side and then outsourced to the cloud. It has become increasingly important to compute encrypted data stored on the remote cloud and untrusted servers due to data outsourcing and increasing user awareness of data privacy [6–8]. In the realm of searchable encryption, the process involves extracting and preserving an encrypted database at the Cloud Service Provider (CSP) and maintaining a local database on the client’s device. A significant challenge arises from the expansion of the local database with the overall growth of the dataset, necessitating increased client storage capacity. Previous research faced complexities in achieving both backward security and maintaining consistent client storage, attributed to clients’ incapacity to transfer data to the CSP, posing potential data leakage risks. Despite existing schemes facilitating retrieval functions on devices, persistent storage overhead remains. Ongoing research focuses on aligning storage overhead with cloud retrieval efficiency while ensuring security, aiming to optimize device performance. The model, denoted as the semi-trusted server, involves the sharing of a database among multiple parties. In this context, certain segments of the data are designated as trusted, while other portions are categorized as untrusted. Servers that are honest but curious should learn as little as possible about SDCs’ data and queries during processing. In the case of this application, an untrusted cloud server is used to store the collections of documents that a client manages using Searchable Symmetric Encryption (SSE). It would also be advantageous if the SDC could query the server and obtain all the records that match their search. This is one of the objectives of the Searchable Symmetric Encryption (SSE) protocol, which is to offer client the ability to carry out those operations while providing the host server precise guarantees about the privacy of the client’s data and queries. This work [9–12] is considered one of the most basic data operations and the research community introduced keywords to search in the ciphertext. Using the static database [13–19], the user uploads their documents to a semi-trusted server for searching and then issues the server search tokens without disclosing sensitive data. Creating, updating, and deleting records are not supported by static SSE.

After this, the researchers turned their attention to Dynamic SSE (DSSE) [20–26]. Secure DSSE schemes are challenging, from a security perspective, because database updates may reveal additional information to the server. Forward and backward privacy has been proposed as an essential security notion for dynamic SE schemes. The forward privacy property [27, 28] prevents a new update from being associated with previous operations (until the keyword matching the existing operation is searched). Many DSSE schemes are not as secure as they should be if an adversary knows something about the added documents, as has been demonstrated by file-injection attacks [29]. To reduce the risk of file-injection attacks [29], forward privacy is crucial. In order for DSSE schemes to avoid such attacks, forward security is essential, meaning that the server cannot find a link between newly added documents and prior search tokens [27]. As a result, we propose a forward-secure DSSE scheme that can achieve these goals.

Another privacy concept, backward privacy property, ensures that if a search is performed for keyword *w* after a document containing *w* is deleted, it does not reveal anything about the deleted document. Although this property was mentioned in [28] neither actual constructions nor a formal definition were presented. Bost et al. [30] provided the first formal definition of the concept of backward privacy, in which three types of leakage were described: Type-I, Type-II, and Type-III.

In an era where privacy concerns are paramount, the demand for secure methods to perform searches over encrypted data has escalated. Building upon this premise, our dynamic DSSE scheme emerges as a robust solution, ensuring not only the confidentiality of sensitive information but also the efficiency required for real-world applications. This paper delves into the intricacies of our proposed scheme, highlighting its unique features and advantages over existing approaches. The computational cost of query generation and the high storage requirements are challenges that most of the current schemes suffer from, as stated in Table 1, and it is still a difficulty that requires a solution for SDC. Constant storage cost for Smart Device Clients (SDCs) in the DSSE scheme addresses the challenge of efficient and secure keyword search on encrypted data, ensuring optimal performance in resource-limited smart devices across diverse applications.

**Table 1:** The comparison of a few (DSSE) structures.

Schemes	Computation		Communication		BP	Client’s storage
	Search	Update	Search	Update		
*Moneta* [26]	$\hat{O}(a_{w}\;logN+{log}^{3}\;N)$	$\hat{O}({log}^{2}N)$	$\hat{O}(a_{w}\;logN+{log}^{3}\;N)$	$\hat{O}({log}^{3}N)$	I	*O*(1)
*Diana*_*del*_ [26]	*O*(*a*_*w*_)	*O*(*loga*_*w*_)	*O*(*n*_*w*_*+n*_*w*_*loga*_*w*_)	*O*(1)	III	*O*(*m*)
*Mitra* [40]	*O*(*a*_*w*_)	*O*(1)	*O*(*n*_*w*_)	*O*(1)	II	*O*(*m*)
*π*_*WBP*_ [39]	*O*(*o′*_*w*_)	*O*(1)	*O*(*n*_*w*_)	*O*(1)	III	*O*(*m*)
*SD*_*a*_ [43]	*O*(*a*_*w*_+*logN*)	*O*(*logN*)	*O*(*a*_*w*_+*logN*)	*O*(*logN*)	II	*O*(1)
*SD*_*d*_ [43]	*O*(*a*_*w*_+*logN*)	*O*(*log*^3^*N*)	*O*(*a*_*w*_+*logN*)	*O*(*log*^3^*N*)	II	*O*(1)
**DBP** [42]	*O*(*a*_*w*_)	*O*(1)	*O*(*n*_*w*_)	*O*(1)	II	*O*(*m*)
**CLOSE-FB** [44]	*O*(*a*_*w*_*+Con*)	*O*(*Con*)	*O*(*a*_*w*_)	*O*(1)	II	*O*(1)
**Our scheme**	*O*(*o′*_*w*_+*LCon*)	*O*(1)	*O*(*o′*_*w*_)	*O*(1)	II	*O*(1)

The above schemes are all forward-private as well as backwards-private. Backward privacy is implied by BP and the number of (keyword, identifier) mappings is indicated by *N*. The number *m* is the number of distinct keywords. Addition operations on *w* are represented by *a*_*w*_,*d*_*w*_ Indicates how many delete operations have been performed on *w*, *o*_*w*_ represents the number of updates on *w* (i.e., *o*_*w*_ = *a*_*w*_ +*d*_*w*_). As at the last search, the number of updates is *o*′_*w*_, where *n*_*w*_ refers to the number of documents that are presently shared with *w*. $\hat{O}$ *m*asks the log logN components. As at the last search, the constant *LCon* represents the total number of computations that can be performed on the hash function.

We propose a scheme based on an inverted index structure presented in pairs (key, value) with control, to retrieve the required encrypted value without needing an SDC-side keyword counter to ensure security, efficiency, and less computational load on the SDC side. Researchers previously faced challenges in achieving constant client storage with backward and forward privacy. Client cannot transfer local storage to the cloud server, which can cause major leakage problems. Thus, we propose an efficient solution to address this issue. For retrieving values from the encrypted inverse index for a specific keyword, the client generates a token based on the keyword and its private key then sends the token to the cloud server. The random value is generated when a keyword is added as a link between the encrypted values of the same keyword in the index, obviating the requirement for an SDC-side keyword counter. The first value of that keyword is extracted from the encrypted index using a token that the SDC gives to the server. With the help of the random value, we can then access the next encrypted value, and so on. In order that the adversary server cannot establish a connection between the keywords that were added and deleted afterwards, the results are returned to the client as encrypted values (backward privacy). As a result, our scheme would efficiently store the constant SDC, as well as low query generation costs, with forward and backward security, as discussed later. The proposed DSSE scheme with constant storage cost for Smart Device Clients (SDCs) holds significant potential in real-world scenarios where privacy-preserving keyword search on encrypted data is vital. Applications include secure cloud storage, healthcare systems, and confidential communication platforms, ensuring data privacy on resource-constrained smart devices.

In summary, the contributions of this work are as follows:

1. Our scheme is the first DSSE framework that is efficient and secure and has a constant storage cost to fulfil the needs of an SDC. Furthermore, our scheme is almost theoretically and practically optimal, with respect to its computational complexity during the search and update processes. As shown in Table 1, it compares well with previous schemes. The scalability of the Dynamic Searchable Symmetric Encryption (DSSE) scheme is pivotal for practical utility in environments with expanding datasets and numerous keywords. Evaluation of our scheme reveals commendable scalability in client storage, as the storage cost remains constant regardless of database size. Computational costs exhibit constancy during query generation, independent of document or keyword numbers, enhancing scalability. Efficient search performance is maintained with a growing database, emphasizing the scheme’s focus on reducing search costs.
2. To the best of our knowledge, our scheme is the only DSSE framework to offer a constant ongoing client storage cost; furthermore, we prove that our system is secure versus semi-trusted adversaries.
3. Using Forward Privacy, new search tokens are generated using a one-time key. It is no longer possible for the server to infer if a previously searched keyword appears in newly added documents by linking previous search tokens with subsequent update tokens.
4. Type-II Backward Privacy. In this case, the server cannot see the update operations (delete or add) during the fulfilling of update queries. SDC-side refinement is performed on deleted documents. Previously searched results are stored on the server in plain text. In this case, re-encrypting such results will not provide any security advantage since they have already been leaked to the server. A further benefit is that plain text results can be read continuously, thereby enhancing the locality.
5. The proposed Dynamic Searchable Symmetric Encryption (DSSE) scheme achieves a remarkable query generation time of 6,460 nanoseconds, ensuring real-time responsiveness and minimal computational overhead. This efficiency, coupled with reduced server-side search costs, enhances user experience, resource utilization, and scalability in applications like secure cloud storage and encrypted messaging systems, showcasing the scheme’s practicality, efficiency, and responsiveness in real-world scenarios.

Our scheme provides keyword generation with low computing overheads ***O***(***o*′**_***w***_**+*LCon***), which is compatible with source-constrained devices.

### A. Paper organization

The remainder of this article is organized in the following manner. Section 2 summarizes related studies. The preliminaries are introduced in Section 3. Section 4 contains the formal definition of symmetric searchable encryption schemes and presents the tools and tail bounds used in our structures and proofs. In Section 5, our experiments and subsequent evaluations are described. A conclusion is provided in Section 6.

## 2 Related work

Song et al. [9] introduced static Searchable Symmetric Encryption (SSE) with a linear search time construction. Curtmola et al. [13] formally defined the security of SSE schemes for the first time. In particular, they developed the first inverted index that kept a list of document identifiers per keyword, which resulted in a sublinear search time. Researchers [16, 18, 31–33] also studied the static case, in which encrypted databases are stored on cloud server where they cannot be changed after they are stored. According to Cash and Tessaro [15], *locality*, space overhead, and *read efficiency* must be balanced. To be clear, rather than just considering read efficiency, they also took into consideration a measure of *overlapping reads*. For this measure, they determined that a scheme has *α overlapping reads* if the reads for a new search overlap at the most *α* bits with all prior reads.

A dynamic version of SSE (DSSE) was then introduced to support database updates, which allows users to add, update and/or remove keywords/document couples at any time within a database [27, 34, 35]. DSSE with sublinear search times was introduced by Kamara et al. [22], but their solution revealed each update’s keyword hashes. The problem was later solved by Kamara and Papamanthou [23], by increasing the complexity of the space. These solutions do not withstand more sophisticated attacks, such as file-injection attacks [29] or leakage-abuse attacks [36]. The attacks reveal the importance of forward private security, an essential property of DSSE that was officially introduced in [28]. While Stefanov et al. [28] presented a forward private, secure DSSE scheme, their solution is complex, since the update protocol will need to be rebuilt at every data structure layer. Some schemes [24, 37, 38] that use Oblivious Random Access Machine (oblivious RAM), can achieve forward security but have high computational costs. Currently, these tools are too expensive for large databases, so they are not practical [39]. There are also schemes [26, 30] with optimal computational complexity and forward security that may suffer from some limitations. The database increases in size by increasing the number of updates (additions or deletions) to the encrypted database that is stored on the server. As the results returned by the server when searching are considered to be a leak *access pattern*, there are not deleted from the server when searching. Storing such information on the server or re-encrypting it will not increase security; rather, it will increase storage costs and subsequent searches. An official definition of forward privacy, together with a DSSE scheme that optimises communication (Sophos) was provided by Bost in [26]. For better theoretical computation complexity than [37], Sophos uses trapdoor permutations to link search and update tokens. Despite this, Sophos is inefficient in practice, as it relies on public key primitives for trapdoor permutation.

An informal mention of backward security was made in [28]. Bost et al. [30] defined backward security in formal terms and gave constructions that realized this property. Two schemes were then proposed by Chamani et al. [40]. The Mitra and Mitra* schemes describe a method that combines the best computational complexity and the best communication complexity, although it requires two rounds of communication to succeed. However, the problem with these schemes is that they suffer from an increase in memory cost at the server and to the client, by increasing the update operations (add/delete) on the data. The symmetric puncturable encryption proposed by Sun et al. [41] improves the puncturable encryption described in [30]. In different adversary models, constructions [39, 42] are provided to enhance privacy; however, the client storage cost is high because it needs data structures that are stored with the clients, such as the keyword counter, whose size increases linearly with the increase in the size of the database. Increasing the cost of client storage was solved in [43, 44]. One limitation is that the cost of search queries increases when searching, which prevents these structures from being used in smart devices.

In contrast from current frameworks, our presented Dynamic Searchable Symmetric Encryption (DSSE) scheme mitigates various constraints by proposing a two-party model that optimizes storage expenses for intelligent devices. Through the strategic utilization of forward and backward privacy measures, the scheme ensures the confidentiality of data during both updates and searches. Notably, the elimination of client-side table’s results in sustained storage, a distinctive feature that significantly enhances operational efficiency. Moreover, the incorporation of secure cryptographic primitives, coupled with a dynamic methodology, facilitates robust privacy-preserving search functionalities. Collectively, these innovations surmount deficiencies in prior schemes, presenting a more effective and secure solution for dynamic searchable symmetric encryption. When it comes to Dynamic Symmetric Searchable Encryption (DSSE), ensuring the dissociation of previous search queries from subsequent requires forward privacy when making updates. While backward privacy guarantees the non-association of subsequent search requests for retrieval of deleted documents from the past. This research introduces a comprehensive method for preserving both forward and backward privacy in DSSE. Impressively, this strategy signifies the first operational and non-interactive Type-II backward-private DSSE framework without relying on secure execution environments. The proposed DSSE scheme attains Type-II backward and forward privacy by generating a unique one-time (fresh) key, denoted as *sk*, for each search query. This yields distinct encryption outcomes for each encryption iteration. Furthermore, it safeguards against the server by discerning the underlying operation (delete or add) embedded in the update query.

Previous research in dynamic searchable symmetric encryption (DSSE) has primarily focused on balancing security and efficiency. ***Mitra*** [40], ***SD***_***d***_ [43], and CLOSE-FB [44] represent efficient schemes in this domain, employing cryptographic symmetric primitives. However, their drawbacks include linear increases in client storage costs and query generation times as the database size grows.

Our proposed scheme distinguishes itself by achieving constant client storage costs and offering heightened efficiency for smart devices. It addresses the limitations of existing schemes, particularly mitigating the linear growth issues observed in ***Mitra*** and ***SD***_***d***_. Through a two-party model and encrypted index implementation, our DSSE scheme not only ensures robust security features, including forward and backward privacy, but also outperforms competitors in terms of search costs and query generation times. This paper provides a comprehensive analysis of the experimental results, substantiating the practical viability and superior performance of our proposed DSSE scheme.

## 3 Preliminaries

### Notation

An $x\overset{\$}{\leftarrow }X$ indicates the uniform selection of ***x*** from a finite set **X**. |**X**| denotes the cardinality of the set **X**. A concatenation operator is used to join strings together, presented by Operator ||. The set {0, 1}^*l*^ represents all binary strings of length *l*, whereas the set {0, 1}* represents all binary strings of a finite length. Our scheme relies on *λ*←ℕ as the security parameter input to all algorithms. During key generation, a uniform sample of a key is taken from the range {0, 1}^*λ*^. In *λ*, we consider only polynomial-time (probabilistic) algorithms and protocols. A particular type of adversary is the Probabilistic Polynomial-Time algorithm (PPT). The bitwise XOR operation between *x* and *y* is represented by the symbol *x*⊕*y*. The proposed method requires the creation of a database **DB**. This **DB** consists of a set of couples of document identifiers and keywords (*ind*, *w*) that appear in the document with the identifier *ind*, when the keyword *w* is present. The term "*negl*" represents an exceedingly diminutive value, approaching a state of virtual non-existence.

The negl(λ) function represents a negligible function negl: ℕ←ℕ in *λ*, in the case of all positive polynomials p(.), where all of them are sufficiently large λ, we have negl(λ)M<1/p(λ). If we use the notation P(c; s) in the context of a client-server system, it indicates that the protocol P is executed by both the client and server based on the input *c* and *s*, respectively.

#### Proofs are based on games

Real and ideal worlds are popular approaches to proving cryptographic scheme security. The real world is used to prove cryptographic constructions and the ideal world is constructed using leakages from the real world. As a result, it is evident that the real world can be considered a secure scheme since the leaked information does not provide any pertinent information to the adversary, as long as no adversary can distinguish between the two worlds. It is anticipated that there are two games in the two worlds: *G*_0_ and *G*_*n*_, and the goal is to ensure that they cannot be distinguished. Unfortunately, this is often difficult to prove directly. The game will instead be built as a hybrid of *G*_1_−*G*_*n*−1_, which will allow each successive game to differ slightly from the one before. Starting from *G*_0_, we prove that it cannot be distinguished from *G*_1_. We continue until we reach *G*_*n*_. An adversary’s advantage in identifying *G*_0_ to *G*_*n*_ is equal to the total partial adversary advantages in identifying (*G*_0_,*G*_1_),(*G*_1_,*G*_2_),… .,(*G*_*n*_−1,*G*_*n*_).

#### Dynamic Searchable Symmetric Encryption (DSSE)

We classify a database **DB** to *n* documents doc = (ind, W_id_). It is mentioned by an identifier ind ⊆ {0,1}^*l*^ and contains a set of keywords W_id_ ⊆ {0,1}*. This indicates that the database DB is defined by ${\left({ind}_{i},{W_{ind}}_{i}\right)}_{i=1}^{n}$ and means it is a list of all the keywords found in the entire $W={⋃}_{i=1}^{n}$, and **DB** (*w*) = {${ind}_{i}|w\in {W_{ind}}_{i}$} is the set of documents that contain the keyword *w*.

As the name suggests, the DSSE scheme ∏ = (Setup,Search,Update) is divided into three parts: Setup, Search, and Update, which requires two parties to participate: a client and a server. There are three parts: two protocols (Update and Search), but only one algorithm, Setup. The client holds the database DB, and the encrypted database EDB is uploaded to the server.

Setup (λ,**DB**; ⊥)→(ϑ,**EDB**) is a setup algorithm. The client creates a state *ϑ* based on a security parameter *λ* and a database. A database encrypted with **EDB** is initialised by the server.

Search(*ϑ*,*w*;**EDB**)→(*ϑ*′,*res*;**EDB**′) is a protocol for undertaking a search. Input to the client is the state *ϑ* and a keyword *w*, whilst the input from the server is the encrypted database **EDB**. Client receives the updated state *ϑ*′ and the search result *res* after this protocol is executed. On the server side, a new update will also be sent to the encrypted **EDB**′ database.

Update(*ϑ*,*in*,*op*;**EDB**)→(*ϑ*′;**EDB**′) is a protocol for updating data. As inputs to the update protocol, the client gets the state *ϑ*, the *in* represented by an inverted index consisted of (*w*, *ind*) pairs and the *op* represented by (*add or delete*), then as outputs of such a protocol, the client gets a newly modified state *ϑ*′ and the server gets the encrypted database-updated **EDB**′.

When a security parameter *λ* and database **DB** are entered, the client executes the algorithm Setup to obtain the secret key *sk*(*K*_*f*_,*K*_*t*_,*K*_*g*_), after which it calls Update protocol *N* times to add the *N* entries of **DB** to **EDB**. For example, in [45, 46] a single build operation is invoked to add the entire database, whereas multiple updates are called. In this case, the search protocol returns only the identifiers of the documents in (*w*). With an additional round, the client receives the actual documents. As shown in [16], DSSE is correct if it can return accurate results for all queries.

#### Security Definitions

The main difference between all existing DSSE schemes is that they leak information at some point. Apart from the schemes based on costly and powerful techniques (e.g., totally homomorphic encryption and oblivious RAM), they leak information at different stages, including how many documents there are in the results [9, 26, 28]. In order to avoid having the adversary learn too much about the database and queries (i.e., search and update), we implement almost all DSSE schemes with some explicit leakage. When the operations Setup, Search, and Update are invoked by the Client, DSSE schemes typically define a leakage function ℒ = (ℒ^*stup*^,ℒ^*srch*^,ℒ^*updt*^) that controls the amount of information exposed to the server.

Standard definitions of DSSE schemes use the real world against ideal world formalizations [13, 16, 22]. A formal definition of games is that there are two types: ${DSSEReal}_{A}^{\Pi }$ and ${DSSEIdeal}_{A}^{\Pi }$. The adversary A discovers no more than the output of leakage functions from a scheme Π if the two games are indistinguishable.

- ${DSSEReal}_{A}^{\Pi }q(\lambda )$. There is honesty in the execution of the DSSE scheme. Similar to the real-world scenario, adversary A is given an **EDB** generated by the Setup (*λ*,**DB**;⊥) protocol. When the adversary selects the keyword w, they receive the transcripts generated as a result of the protocol Search(*ϑ* ,*w*;**EDB**). Furthermore, the adversary can select an *in* set of pairs (*w*,*ind*) and an operation *op*, to receive the duplicates generated from the Update(*ϑ*,*in*,*op*;**EDB**) protocol. An adversary eventually outputs bit*b* ∈{0,1}.
- ${DSSEIdeal}_{A}^{\Pi }q(\lambda )$. The adversary receives a simulated transcript as a substitute for the real protocol transcripts. A leakage function with known outputs can be used; a PPT simulator S generates the simulated transcripts. The adversary receives an encrypted database generated by $S(L^{Setup(q_{i})})$. As the adversary performs search and update operations, it receives transcripts generated by $S(L^{srch(q_{i})})$ and $S(L^{srch(q_{i})})$. An adversary eventually outputs bit *b* ∈{0,1}.

**Definition 1**. (DSSE Scheme [28]). A DSSE scheme ⊓ with leakage function ℒ is adaptively secure if, for every PPT adversary A, the number of queries *q*(*λ*) issued is polynomial. Simulator S satisfies the following requirements:

$$\left|pr[{DSSEReal}_{A}^{\Pi }q(\lambda )=1]-pr[{DSSEIdeal}_{A,S,L}^{\Pi }q(\lambda )=1]|≼negl(\lambda \right)$$

#### Common Leakage

The first step is to realize common leakage functions, as in [30]. The internal state of leakage functions is determined by query set *Q*, which includes an entry for every query issued. In *Q*, each entry represents a pair (*t*,*w*). This search query uses the keyword *w* and a counter *t*, which starts at 0 and increases with each query. The search pattern *sp*(*w*) corresponds to each keyword w defined in [26].


sp(w)={t|(t,w)∈Q}

SSE schemes that provide efficient security [13, 26, 28, 30] allow leakage known as *access pattern* [13], which contains the set of documents matching the keyword in the search operation.

Here, we used the same notation as in [26], HistDB(w). This consists of a list of documents containing keywords *w* that were historically added to the database, in the order in which they were inserted. The document identifiers are included in the list once the documents are added, regardless of whether they have been deleted or not. When HistDB(w) = {*ind*_1_,*ind*_2_}, it means that *ind*_1_ and *ind*_2_ were historically added to **DB**, but it is not known if they have been removed. For a keyword *w*, the function called TimeDB(w) takes the timestamp/document-identifier pairs that have not been deleted from the database yet.


$$TimeDB(w)=\{(t,ind)|(t,add,(w,ind))\in Q,∄t^{\prime },(t^{\prime },del,(w,ind))\in Q\}.$$

For all updates associated with w in Q, the timestamps are expressed as follows:

Updates(w)={t|(t,add,(w,ind))∈Q⋁(t,del,(w,ind))∈Q}.

#### Forward Privacy

With forward privacy, earlier search queries are guaranteed to be unrelated to the current update. We can use this feature to hide whether a specific keyword is associated with the recent addition operation.

**Definition 2** (Forward Privacy [26]). An ℒ-adaptively-secure DSSE scheme, the forward private keyword supports update operations on a single keyword iff ℒ^*stup*^, ℒ^*srch*^, and ℒ^*updt*^ as described below:

$$L^{stup}=\emptyset ,\;L^{srch}(w)=\{sp(w),HistDB(w)\},\;L^{updt}(op,w,ind)=L^{\prime updt}(op,ind)$$

In this case, ℒ′ indicates a stateless function.

#### Backward Privacy

When providing a search query for a keyword *w*, in which some of its entries have been deleted, backward privacy minimises the amount of information that will be leaked to the server. When (*w*, *ind*) is added to the database and subsequently removed, the DSSE scheme is considered backward-private. The search query only reveals *ind* when released, after adding (*w*, *ind*) and before deleting it. Bost et al. [30] defined backward privacy as being of Type-I, Type II, or Type III. Type-I provides the lowest level of information and Type-III provides the most information.

**Definition 3** (Backward Privacy [30]). An ℒ = (ℒ^*stup*^, ℒ^*srch*^, and ℒ^*updt*^) adaptively-secure DSSE scheme ⊓ with backward privacy, iff ℒ is described as follows:

$$L^{stup}=\emptyset ,\;L^{srch}(w)=L^{\prime \prime srch}\{TimeDB(w),Updates(w)\},\;L^{updt}(op,w,ind)=L^{\prime updt}(op,ind)$$

In this case, ℒ′ and ℒ″ indicate a stateless function.

#### Locality and Read Efficiency

Based on Cash and Tessaro [15], we define locality and read efficiency.

**Locality:** SSE schemes can be categorised based on the number of contiguous reads from the encrypted database EDB. Specifically, if we assume that the Search algorithm only gets access to the encrypted database through the Oracle database, Oracle returns words stored in EDB intervals that correspond to the query intervals [*a*_*i*_; *b*_*i*_]. By using the Search algorithm, the oracle is queried for some interval [*a*_*i*_; *b*_*i*_], using a token as a basis. Based on *τ* and previously read intervals, it continues to calculate the next interval to be read from EDB. The intervals are indicated by ReadPat(**EDB**;τ).

**Definition 4** (*Locality*). There is a locality in a searchable encryption scheme *r*-local if, for any *w*∈*W*, RdPat(w) contains at most *r* intervals. It should be noted that, Localities are perfect when r = 1.

**Read efficiency:** A single interval can read the entire database within the Search algorithm, so there is no concept of a lone locality. Read efficiency measures the amount of data read by a search operation. Considering a given *DB* and *w*, we can let |*DB*(*w*)| represent the issue of words contained within the encoding of *DB*(*w*).

**Definition 5** (*Read Efficiency*). A searchable encryption SE scheme has c-read efficiency if, for any *w*∈W, the total number of bits of **RdPat**(*w*) is a maximum of *c*.|*l*.*DB*(*w*)|bits, where *l* is the issue of bits required to enable one document identifier.

**Definition 6** (*Overlapping Reads*). A searchable encryption SE scheme has *α*-overlapping reads if the reading patterns (of all keywords) are overlapped with mainly *α* bits. In the case of *α* = 0, we have disjointed readings.

## 4 System model overview

This section presents our forward-backward privacy scheme. We developed an ongoing SDC storage cost, forward privacy, and backward privacy scheme based on Type-II definitions. This prevents subsequent searches from being able to relate to the deleted documents. According to the proposed scheme, a two-party model is used, where the client is the owner of the data, and the server is the one that provides large storage space and efficient computational power to the client.

A scheme comprises one algorithm setup and two protocols for updating and searching. In the setup algorithm, the client initializes specific parameters and structures used in other protocols to enable the DSSE system to operate correctly. The client encodes keywords/document couples for subsequent searches in the Update protocol. Based on the current global counter *Con*, an **EDB** is built for every keyword *w* in the update. The search token *KT*_*w*_ is generated by the client and sent to the server based on the keyword *w* and the number of global users. The search token can be used: the server produces all necessary addresses for keyword *w* and looks for documents in **EDB**, which are stored under keyword w.

**Construction of proposed scheme.** *GenPRP*(1^*λ*^),*GenPRF*(1^*λ*^)—a key is generated through this process. Functions, F:{0,1}^*λ*^ X{0,1}*→ {0,1}^*λ*^ is a pseudo-random function *PRF*, *h*:{0,1}*→ {0,1}^*λ*^ describes a hash function in the form of a random oracle, and *G*: {0,1}^*λ*^X{0,1}^*λ*^→ {0,1}^*λ*^ is a pseudo-random permutation function *PRP*. We describe our scheme in one algorithm and two protocols.

**Setup.** *K*_*f*_, *K*_*t*_, and *K*_*g*_ represent the keys generated randomly by the **Setup** algorithm1; we formally describe each with a size determined by the security parameter *λ*. As a long-term key, *K*_*f*_ prevents a valid search token from being generated by the server in *PRF F*, *PRP G* uses *K*_*g*_ to protect pairs of (*ind*, *op*), while *K*_*t*_ constructs tags for keywords *w*. A constant and minimal amount of storage is required on the client side for the global counter *Con* (used with a search token for generating the locations *Addr*), and the client initiates two empty maps (*S*_*e*_, *S*_*r*_), which are sent to the server for storage. In *Se*, encrypted entries are stored for each call update protocol, while in *S*_*r*_, plaintext entries are stored after the search operation is finished.

**Update.** As described in algorithm 2, it is important to remember that our scheme does not apply to a single keyword but to a document or set of documents. Therefore, the keyword index must be extracted from algorithm2 lines (1–4), which is self-evident in all efficient schemes that deal with the index directly without addressing its extraction. Clients in update protocols provide document collection *DocColl* and an update operation *op*, such as adding or deleting documents. A local state *ϑ* contains information about the keyword *w*, three keys (*K*_*g*_,*K*_*f*_,*K*_*t*_), and the system constant *Con*. Adding a document set *Doc* to an encrypted database *Se* involves adding a keyword/document (*w*,*ind*) pair for every document in the set. A document is first taken out of the client lines (1–4) from algorithm2; in this step, keywords are extracted from each file in the collection (the keyword plus the documents it contains) indexing. This step is considered essential in all systems. Before adding or deleting keywords, the index is extracted. Therefore, it is the index that is dealt with, rather than the document directly. The extracted index is sent to a procedure called Process. The Process procedure works as follows:

When executing *PRF F*, the client uses *K*_*f*_ to calculate ${KT}_{w}\leftarrow F_{K_{f}}(w_{i})$ for each keyword *w* in index *KTMap*.*KT*_*w*_ is included in the calculation of the search token *Key*_*w*_, which is combined with the system constant *Con* hash function; with each update, the value decreases. It is necessary to ensure that it does not conflict with the previous update, the forward privacy. *Key*_*w*_ is used to calculate the locations where encrypted values will reside in the server’s *S*_*e*_ Hash table. The location of the subsequent value is calculated using the random value *rn*, that is randomly generated for each pair(*w*,*ind*), except for the last couple in the index for the base word. An empty value ⊥ is incorporated as the index’s end tag in the subsequent search process. Next, the couples are encrypted (*w*,*ind*) to prevent them from being explicitly revealed to the server (backward privacy). A masked value *Val* is obtained by XORing the second outcome H(*Key*_*w*_||0)with the encrypted entry *E*_id_. These steps are repeated for all keywords in the document. Every time an update request is made, the Fresh Key *sk* is computed, so that the highest levels of privacy and security are achieved. During the update, this key is used to encrypt value (*ind*||*op*) to hide it from the server, as well as to prevent it from getting leaked.

**Fig 1:**
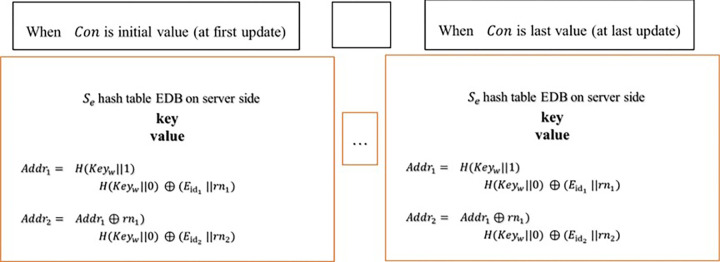
Updates on keywords ***w***_1_.

A hash table is a data structure that stores information in a (key, value) format, allowing direct access to elements in a constant time. The innovative approach involves storing the *rn* value to calculate the next location for the same keyword within the current cell, ensuring that location calculation and retrieval tasks remain on the server for faster retrieval of results while maintaining data confidentiality. (Fig 1 illustrates this concept.)

When the **process** is complete, the system constant (*Con*−1) at the client is updated and the $S_{e}^{\prime }$ is added to the encrypted index *S*_*e*_ at the server. In Fig 1, the keyword *w*_1_ is updated for a few file collections.

**Search.** Algorithm 3 formally describes the search. The algorithm receives input from both the client, denoted as ’ϑ,’ and the associated keyword. Additionally, it takes into account the contents of the server, encompassing both the encrypted *S*_*e*_ and explicit *S*_*r*_ databases.The client establishes a location within the explicit database, assigning it the value *t*_*w*_, and concurrently generates a private key *KT*_*w*_ specifically for the keyword *w* lines (1–4) in algorthim3. At the start, the clients provide a tag value *t*_*w*_, so that the server can return the document identifiers and the last constant *Lcon* value (if it exists) for *w* based on the most recently completed search request. Depending on the *access pattern* leakage, the explicit data will be leaked to the adversary, so it is stored explicitly in a hash table *S*_*r*_[*t*_*w*_] at the server after each search query. By hashing the keyword search token ${KT}_{w}\leftarrow F_{K_{f}}(w)$ For *Con* times, the server calculates the locations for searching the encrypted database EDB *S*_*e*_. Whenever the current *Con* equals the last constant *Lcon* stored in *S*_*r*_[*t*_*w*_], the search process terminates because no updates have occurred since the previous search. Otherwise, a set of locations *Addr*_*w*_ is computed for encrypted values in *w*, with each *Con* value between the last update and the last search. Consequently, the server calculates the location *Addr*_*w*_ for each encrypted value. Subsequently, the encrypted value for that specific location is positioned within the *ID*_2_ group. Following this placement, the retrieved encrypted value is removed through the action of deleting (Delete *S*_*e*_[*Addr*_*w*_]) from the encrypted index. The value of the location *Addr*_*w*_ is calculated each time, with the help of the random number *rn* stored in the encrypted value of the previous location until the last value contains *null*, as shown in Fig 1. The steps (lines 11–22 in algorthim3) are repeated until the current constant *Con* number equals the last regular *LCon* number. The encrypted results, put in *ID*_2_, are transmitted to the client, which possesses the requisite private keys for decrypting the results obtained from the server. Algorithm 3, specifically lines (25–27), is employed for the decryption process. Subsequently, the client generates a filter line (25–27) to identify and retain the values that were added, ensuring that only those additions and not the deleted values persist within the designated group *ID*_1_. After decryption, the plaintext data and current *Con* are returned to the server for storage in the hash table *S*_*r*_. This arises due to its classification as an access pattern leak. Re-encrypting it would result in an elevated computational cost during both encryption and decryption processes when subsequently conducting a search for the associated keyword w.

Alogorithm1 **Setup**(*Con*,*λ*;⊥)

  **Client:**

1: $K_{g}\overset{\$}{\leftarrow }GenPRP(1^{\lambda }),{\{K}_{f},K_{t}\}\overset{\$}{\leftarrow }GenPRF(1^{\lambda })$ // Generate encryption and decryption keys

2: *Con*←*System Constant* // Give an initial value

3: *ϑ*←(*K*_*g*_,*K*_*f*_,*K*_*t*_,*Con*) to the **client** side

4: *S*_*e*_,*S*_*r*_←*empty map* // Create two empty explicit and encrypted indexes

 Send *S*_*e*_, *S*_*r*_ to the **server side**.

Algorithm 2 **Update**(*ϑ*,*DocColl*,*op*; *S*_*e*_)

 **Client:**

1: *KTMap* ←*MultiMap* // create multimap: is contains a sorted list of key

2: *for i* = 1 *to*|*DocColl*| // Iterate over the number of documents in the collection

3: *for j* = 1 *to* |*Doc*_*i*_| // Iterate over the number of keyword w in the document

4: *KTMap*[*w*_*i*_]←*KTMap*[*w*_*i*_]⋃{*ind*} // Collect IDs for documents that contain keyword w

5: $S_{e}^{\prime }\leftarrow Process(\vartheta ,op,KTMap)$ // call function

6: Upgrade *ϑ*←(*K*_*g*_,*K*_*f*_,*K*_*t*_,*Con*−1) // Update client state content

  **Server:**

7: Add $S_{e}^{\prime }$ To *S*_*e*_ // Update the encrypted index

ProcedureProcess(ϑ,op,KTMap)

1: (*K*_*g*_,*K*_*f*_,*K*_*t*_,*Con*)←*ϑ* // Client content

2: *Temp*←*empty map* // Create empty group

3: *for i* = 1 *to* |*KTMap*| // Iterate along the explicit index

4: ${KT}_{w}\leftarrow F_{K_{f}}(w_{i})$ // Create a key associated with the keyword w for first ind

5: *Key*_*w*_←*H*(*KT*_*w*_||*Con*) // Create fresh key for first ind

6: *Addr*_*w*_←*H*(*Key*_*w*_||1) // Calculate locations encrypted values for first ind

7: *for j* = 1 *to* |*KTMap*[*w*_*i*_]|−1 // Iterate the number of ind that contain the keyword

8:  $rn\overset{\$}{\leftarrow }{\left\{0,1\right\}}^{\lambda }$ // Create random value *rn*

9:  $sk=F_{K_{g}}(w||con)$ // Create an encryption key for that value to be encrypted

10: *E*_*id*_←*G*_*sk*_(*ind*||*op*) // Encrypt the identifier and operation with a permutation function

10:  Val ←*H*(*Key*_*w*_||0) ⊕(*E*_*id*_||*con*||*rn*) // Creating the ultimate encrypted value

11: *Temp*[*Addr*_*w*_]←Val // Store the encrypted value in the temporary index

12:  *Add*_*rw*_←*Add*_*rw*_⊕*rn* // Combine the *rn* with the current location to calculate a new location

13: *E*_*id*_←*G*_*sk*_(*ind*||*op*) // Encrypt the identifier and operation with a permutation function for last ind

14:  Val ←*H*(*Key*_*w*_||0) ⊕(*E*_*id*_||*null*) // Creating the ultimate encrypted value for last ind

15: *Temp*[*Addr*_*w*_]←Val // Store the encrypted value in the temporary index for last ind

16: Return *Temp*

Algorithm 3 ***Search***(*ϑ*, *w*;*S*_*e*_,*S*_*r*_)

**Client:**

1: (*K*_*g*_,*K*_*f*_,*K*_*t*_,*Con*)←*ϑ* // Client content

2: *ID*_1_,*ID*_2_←*empty set* // Create two empty groups

3: $t_{w}\leftarrow F_{K_{t}}[w]$ // Create an location within the explicit database

4: ${KT}_{w}\leftarrow F_{K_{f}}(w)$ // Create a private key for the keyword w

5: Send *t*_*w*_,*KT*_*w*_ to the **server side**.

**Server:**

6: **If** *S*_*r*_[*t*_*w*_] ≠*null* // explicit database contains values for keyword w

7: (*ID*||*con*)←*S*_*r*_[*t*_*w*_] // Retrieve plaintext previous search values

8:  *Lcon*←*con* // Retrieve the constant system of the last search

9: **Else**

10: *Lcon*←*System Constant* // If there was no previous search for the keyword w

11: *for i* = *Con to Lcon* // Iterate over a constant system

12:  *Key*_*w*_←*H*(*KT*_*w*_||*i*) // Retrieve fresh key

13:  *Addr*_*w*_←*H*(*Key*_*w*_||1) // Calculate locations encrypted values

14:  **If** *S*_*e*_[*Addr*_*w*_]≠*null* // If this location contains an encrypted value

15:   (*E*_*id*_||*i*||*rn*)←*S*_*e*_[*Addr*_*w*_]⊕*H*(*Key*_*w*_||0) // Retrieve encrypted value

16:   *ID*_2_←*ID*_2_⋃{*E*_*id*_||*i*} // Put the encrypted values into a group *ID*_2_

17:   Delete *S*_*e*_[*Addr*_*w*_] // Delete the retrieved encrypted values from the database

18:   *While rn* ≠ *null* // Repeat until variable *rn* equals *null*

19:    *Add*_*rw*_←*Add*_*rw*_⊕*rn||rn* use to calculate the next location for the same w

20:  (*E*_*id*_||*i*||*rn*)←*S*_*e*_[*Addr*_*w*_]⊕*H*(*Key*_*w*_||0) // Retrieve encrypted value

21:   *ID*_2_←*ID*_2_⋃{*E*_*id*_||*i*} // Put the encrypted values into a group *ID*_2_

22:    Delete *S*_*e*_[*Addr*_*w*_] // Delete the retrieved encrypted values from the database

23: Send *ID*_1_,*ID*_2_ to the **client side**.

     **Client:**

24: *for i* = 1 to |*ID*_2_| // Iterate the number of encrypted values retrieved

25:   (*E*_id_||*number*)←*ID*_2_[*i*] // Separating the variable *number* from the encrypted values

26:   $sk=F_{K_{g}}(w||number)$ // This variable goes into calculating the decryption key

27:  $\left(ind||op)\leftarrow G_{sk}^{-1}(E_{id}\right)$ // Using the permutation function, the key returns the plaintext

28:   **If** *op* == "*add*" / A filter will be created for deleted values

29:    *ID*_1_←*ID*_1_ ⋃ {*ind*} // The remaining values are only added

30: Send *ID*_1_,*Con*,*t*_*w*_ to the **client side**.

  **Server:**

31: *S*_*r*_[*t*_*w*_]←(*ID*_1_||*Con*) // The plaintext results of the current search are stored on the server

Subsequent to each query on the keyword set denoted as *w*, a deduction of one is made from the constant value (*Con*−1) to produce a one-time key (*sk*​) specific to *w*. This sequential process serves to deactivate previously exposed keys. Consequently, when the keyword *w* is incorporated into a new document, its corresponding entry undergoes safeguarding through the assignment of a fresh key. This preventive measure precludes the server from establishing connections with prior issued search queries, thereby substantiating the concept of forward privacy. An instruction is given to the server to expunge all pertinent entries from the index in alignment with this security protocol.

### A. Security analysis

In this section, we analyze our scheme’s security. The security proofs within the random oracle model establish a robust foundation for affirming the privacy-preserving attributes of the DSSE scheme. Leveraging game-based analysis and the random oracle model instills confidence in the scheme’s security, signaling resilience against potential adversarial attacks. In essence, the security proofs within the random oracle model provide a systematic and formal exhibition of the DSSE scheme’s resilience to diverse adversarial scenarios, reinforcing its assertions of Type-II backward and forward privacy. These proofs significantly enhance the overall credibility and trustworthiness of the proposed DSSE scheme in real-world cryptographic applications.

As a result, we achieve Type-II backward and forward privacy under our strategy, as shown in the theorem below.

***Theorem* 1**: *A DSSE scheme with forward and backward privacy is a scheme with F modeled as a secure PRF, G as a secure PRP, and h as the hash function, with the leakage defined as follows*:

$$L^{stup}=\emptyset ,\;L^{srch}(w)=L^{\prime \prime srch}\{TimeDB(w),Updates(w),sp(w)\},\;L^{updt}(op,w,ind)=\emptyset$$

**Proof**: We prove it using a hybrid argument that involves a series of games. Games *G*_0_ and *G*_4_ are identical to real-world SSE games in the ideal world and real-world SSE games in the real world.

Game *G*_0_. This game simulates SSE security in the real world. This results in:

$$P[{DSSEReal}_{A}^{\Pi }q(\lambda )=1]-P[G_{0}=1]=0.$$

Game *G*_1_. This is essentially the same as *G*_0_ but the hybrid does not use the function *F* to generate *KT* and *t*_*w*_; random numbers are used instead. To bind to a keyword *w*, KWT and TWT are two tables used to store an arbitrary string linked to the keyword *w*. More specifically, when dealing with a new keyword *w*, the system picks a unique random string and stores it in two tables (*KWT* and *TWT*) so that it can be re-used when *w* is queried. It is possible to distinguish *F* from a real random function if an adversary can distinguish *G*_1_ from *G*_0_. This means we can build an adversary $A_{1}$ that is as efficient as:

$$P[G_{0}=1]-P[G_{1}=1]\leq 2.{Adv}_{A_{1},F}^{prf}(\lambda )\leq negl(\lambda )$$

Game *G*_2_. The output of function *G* is simulated using the table *GT* in this hybrid. The hybrid miscarry is caused if the output appears in more than one entry in *GT*. The likelihood of such an occurrence is negligible. Again, to distinguish *G*_1_ from *G*_2_, an adversary $A_{2}$ should be able to distinguish between *G*, concerning a randomly generated function, i.e.


$$P[G_{1}=1]-P[G_{2}=1]\leq {Adv}_{A_{2},G}^{prp}(\lambda )\leq negl(\lambda )$$

Game *G*_3_. In this hybrid, to answer the random Oracle query, we create three tables, HashT_1_,HashT_2_, and HashT_3_, each of which records the response to the *H*(*KT*_*w*_||*Con*) and the *H*(*Key*_*w*_||0) and *H*(*Key*_*w*_||1). If we only consider the addition process, when considering leakage in the Update function, we define ℒ^*updt*^ = |DB(*w*)|, which will leak the total number of keyword/document couples. Instead of calling hash function *H* for *Con* times, the **Process** protocol generates the search token *Key*_*w*_ with a random string. As part of the process of generating tokens, the hash functions *h*_1_ = *H*(*Key*_*w*_||0) and *h*_2_ = *H*(*Key*_*w*_||1) are replaced with random strings. As a result, the random oracle H is programmed such that, in the protocol for searching the data, *H*(*KT*_*w*_||*Con*) = *Key*_*w*_,*H*(*Key*_*w*_||0) = *h*_1_, and *H*(*Key*_*w*_||1) = *h*_2_. We keep track of transcripts each time the hash function is called through tables HashT_1_,HashT_2_, and HashT_3_. It will be possible to distinguish *H* from a real random function if an adversary can differentiate *G*_3_ from *G*_2_. As a formal construction, we can create an adversary $A_{3}$ that is efficient in the following ways:

$$P[G_{2}=1]-P[G_{3}=1]\leq {Adv}_{A_{3},H}^{hash}(\lambda )\leq negl(\lambda )$$

Game *G*_4_. An **Updates** table is created, indexed by keyword *w*, and maintained in this hybrid for generating search tokens. The search token *Key*_*w*_ is generated by *G*_3_. by hashing *Con* times rather than using an existing token. As an alternative to mapping the keyword to the value picked from EDB *S*_*e*_, we use the immediate ‘Update’ table that maps the keyword directly to the update counter. This gives the following:

$$P[G_{3}=1]-P[G_{4}=1]=0$$

Simulator. As a final step, we generate a simulator S that utilises the leakage function ℒ to produce the ideal game, ${DSSEIdeal}_{A,S,L}^{\Pi }q(\lambda )$, which is similar to game *G*_4_. S does not recognise *w*, which is the only difference. Instead, it relies on the leakage function’s $\hat{w}\leftarrow min\;sp(w)$. As a result, we have the following:

$$P[G_{4}=1]-P[{DSSEIdeal}_{A,S,L}^{\Pi }q(\lambda )=1]=0$$

The following result is obtained by combining the results of all the games mentioned above:

$$P[{DSSEReal}_{A}^{\Pi }q(\lambda )=1]-P[{DSSEIdeal}_{A,S,L}^{\Pi }q(\lambda )=1]\leq 2.{Adv}_{A_{1},F}^{prf}(\lambda )+{Adv}_{A_{2},G}^{prp}(\lambda )+{Adv}_{A_{3},H}^{hash}(\lambda )\leq negl(\lambda )$$

*Conclusion*: The simulator should use random strings instead of *ind*. The EDB requires new search tokens for each new entry, each time it is updated. As a result, the server cannot distinguish random entries from those observed during the update. Furthermore, the server has no idea what kind of operation it is performing. It only leaks the number of entries through its update protocol. To guarantee backward private security, the server knows how many entries are related to keyword *w* and when each update happens. Server cannot learn anything more from the search protocol. As a result, the server cannot determine which deletions delete which additions.

Concerns encompass an adversary with background knowledge, cryptographic primitive assumptions, reliance on the random oracle model, susceptibility to quantum attacks, assumptions about no collusion, a limited leakage model, dynamic changes in system parameters, and considerations regarding scalability and efficiency. These factors underscore the importance of a nuanced evaluation, considering real-world scenarios, potential weaknesses in cryptographic primitives, quantum computing advancements, collusion scenarios, leakage during updates, dynamic system parameter changes, and scalability concerns for practical applicability.

## 5 Evaluation

In this section, our scheme is evaluated and compared with related schemes. Java is the programming language used in all schemes. Cryptographic hash functions *h* are performed with SHA-256 (160 bits long). *PRF F* is released with AES-128/256, while *PRP G* is released with AES-128/128. The experiments were conducted on a HUAWEI laptop running Windows 11 64-bit, x64-based operating system, using an Intel(R) Core (TM) i5-10210U CPU at 1.60GHz 2.11GHz, 8GB of RAM, and 512GB SSD disk.

The data set [47] consists of a large set of text e-mail messages exchanged between Enron employees. The data set is distinguished by its large size, which provides a diverse and realistic sample for testing information retrieval systems. The Enron email database serves as the foundation for document extraction and keyword processing. Each email file within the dataset is treated as a document, considering metadata and content. During the setup phase, keywords indicative of document content are extracted and subsequently encrypted using cryptographic functions such as pseudo-random functions (PRF) and pseudo-random permutations (PRP). The DSSE’s setup algorithm initializes critical parameters, generates cryptographic keys (*K*_*f*_,*K*_*t*_,*K*_*g*_), and establishes the encrypted database (EDB) structure, mapping keywords to respective documents. Client-side storage encompasses essential information, including cryptographic keys and a global counter (*Con*), while maintaining data structures (*S*_*e*_,*S*_*r*_) in server side.

In these experiments, all document-keyword pairs in the Enron email dataset [47] were extracted and stored in an EDB. Variable size of the result was constructed throughout the experiment, ranging from 10 to 10^5^ documents. To simulate the performance impact of deletions on update operations, we simulated deletions with a probability *ρ* of 0.1. In the simulation of deletion processes during the system’s beta testing, a dynamic environment is established to replicate real-world scenarios where documents are systematically removed over time. Within the experimental framework, a deletion process involving pairs from the same database is incorporated at a rate of 10 percent. Specifically, for every addition of nine pairs (keyword, identifier), the tenth pair introduced corresponds to the removal of one of the preceding nine pairs, thus simulating the ongoing evolution of the database. When the time for processing each entry was calculated by repeating the experiment 30 times and then taking the average of the results; more entries were processed, the processing time for each entry decreased. The searchability of all keywords was preserved, even those that were misspelled or typed incorrectly. In addition, the file size did not affect search costs; it affected insertion costs, as bigger files typically include more keywords.

The proposed DSSE scheme undergoes a comprehensive evaluation, addressing client storage, computation cost, dynamic operation effects, and decryption cost on the client’s side. Focusing on efficiency as the database size expands; client storage assessment seeks a constant cost for smart devices with limited local storage. Computation cost measures query generation efficiency, emphasizing lower costs for enhanced scheme performance. Dynamic operation effects simulate the scheme’s behavior during updates and searches under dynamic scenarios in real world systems. Decryption cost evaluation ensures efficient result decryption, crucial for backward privacy in dynamic schemes. These metrics collectively offer a thorough assessment, aligning with key considerations in designing secure and efficient DSSE schemes, particularly in scenarios involving smart devices and dynamic data updates.

A comparison can be made between the proposed scheme and efficient schemes *Mitra* [40], *SD*_*d*_ [43] and CLOSE−FB [44]. Cryptographic symmetric primitives are used in all of the compared schemes, allowing for a fair comparison. For the simulation of dynamic search and update queries on a particular keyword, we created a sequence Seq of 100,000 queries that contained both the search and the update, interleaved by each other. As part of the construction of this sequence, a search parameter *ρ* was used to determine the probability of a search query appearing in the series, i.e. (1- *ρ*) is the probability of an update query occurring.

One of the criteria for selecting a variable-sized result is the quantity of documents containing the searched keyword and the associated deletion operations. Notably, in our proposed scheme, the addition and search operations are retained on the server to uphold data privacy.

### A. Client storage

**Fig 2:**
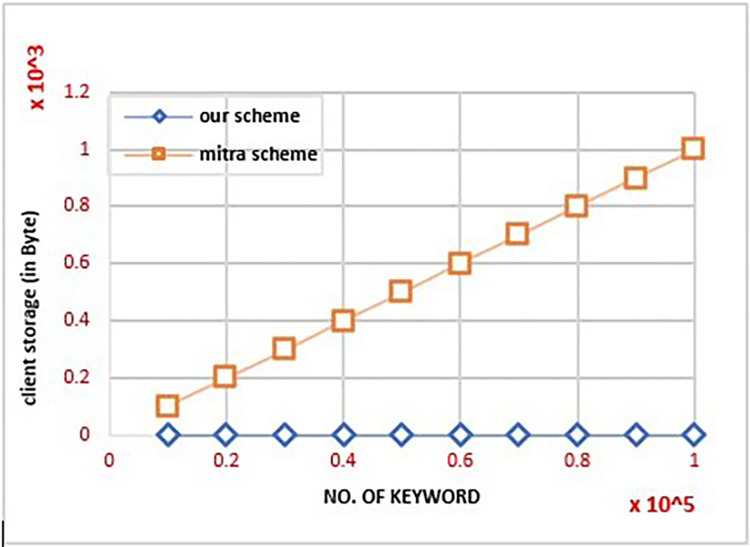
The client cost of storage by increasing the size of the database.

One of the characteristics of smart devices that make them successful is that local storage is constant. This experiment compared our scheme with some efficient related schemes, such as the Mitra scheme, in terms of cost and hold on the client’s side. A counter *a*_*w*_ counted the number of updates required local to the client, for each keyword in the dataset. Almost all efficient schemes store the keyword update and search counter in one or more tables with the client, resulting in a higher storage cost. Thus, the cost of storage at the client end increases linearly, as the size of the database increases. Our scheme does not require these tables because the client only has the keys, and so storage is constant. In our scheme the bulk of the processing and storage responsibilities are moved to the server side. As a result of the fact that storage at the client remains constant, even as the database size grows, the proposed work demonstrates high efficiency in this experiment. The client’s storage cost is shown in Fig 2. We did not include the *SD*_*d*_ [43] schemes in Fig 2 because it also has a constant storage cost from the client. Unfortunately, for both *SD*_*d*_ and *Mitra*, the query generation time increases linearly with the increase in the number of results associated with it and the volume of the accumulated increase in EDB, as shown in the following experiments.

### B. Computation cost

**Fig 3:**
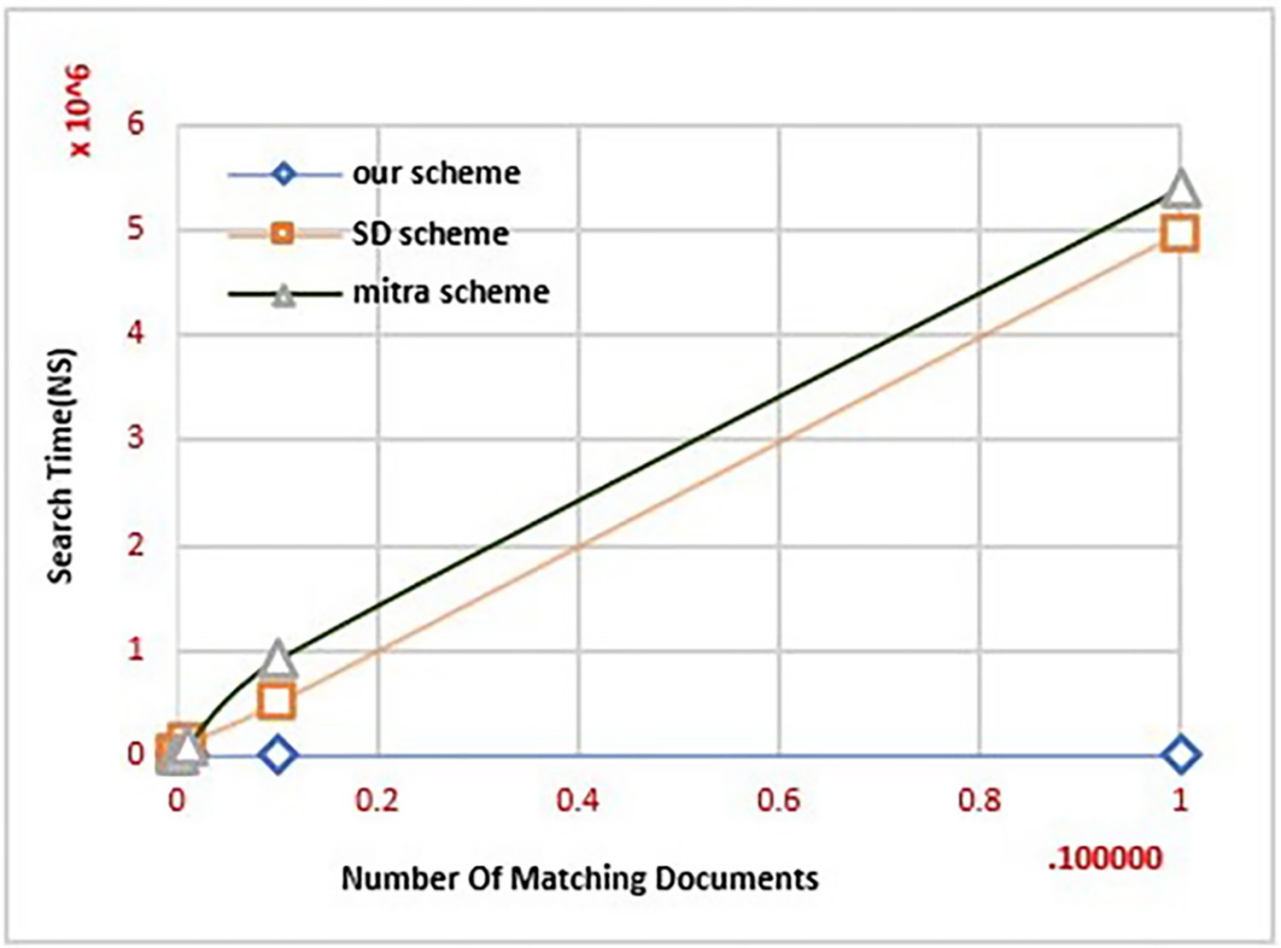
The generation time of the query.

We compared our proposed scheme against other schemes, to see the computation costs for the client. The query generation of the searching keyword is shown in Fig 3, as a function of the number of matched documents to this keyword. When a keyword is required, there is a lower computation cost for the client. When searching for a keyword, we only need to calculate *KT*_*w*_ once (in every search, it is considered a fresh key), regardless of the number of documents containing the keyword. In other efficient schemes, *SD*_*d*_ and Mitra, the computational cost of query generation to the client increases linearly, with the document number in the encrypted index associated with that keyword. Because they need to calculate all the locations associated with the keyword *w*, the account cost will be increased for the client. In this way, we can demonstrate that our scheme works efficiently with smart devices.

### C. A dynamic operation’s effect

**Fig 4:**
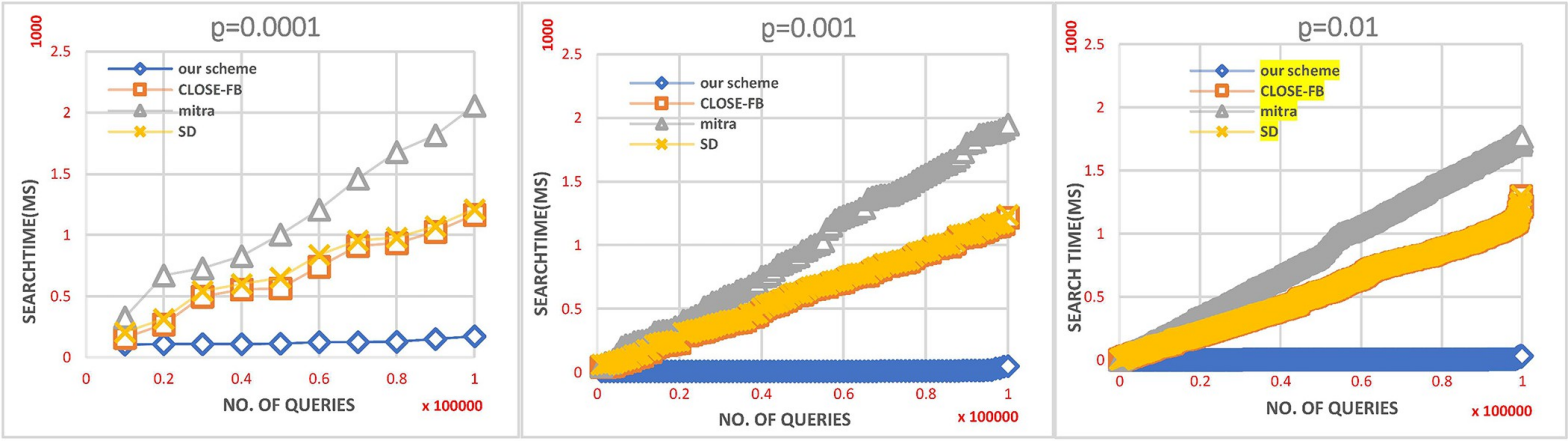
Search performance in dynamic mode.

A simulation of the performance of search queries under a dynamic environment is presented in this section. A simulation was also conducted using traces to determine whether this improvement was significant. This experiment embodies a practical application of encrypted data retrieval systems in a real-world scenario, wherein three distinct traces were generated. Each trace incorporates updates, encompassing additions and deletions, along with searches for specific keywords. We set the trace length to 100,000 results and the probability of each search query was stored in each trace as a parameter *ρ*. There is a possibility *ρ* that the query in the tracking is a search query. Since these are dynamic systems, a specific keyword is added, and over time, the same keyword is searched for when it is needed. In contrast, the probability of an update query is 1 – *ρ*. A client was allowed to replay the traces during the experiment and we found this to be a very accurate simulation of a real-world situation, where updates and searches are mixed. Using the trace below, we recorded the time each search query took on the server side (between the time of receipt of the search token and the time of receipt of the results). The search time for Sequence queries was accumulated on the server side during this experiment. An illustration of the search time for various values can be seen in Fig 4. A linear relationship exists between the number of update queries executed and the search time for Mitra and CLOSE-FB. DSSE schemes must read additional entries for every query due to their high *locality*. Therefore, plaintext results are more localised since they can be read continuously. Previous research results considered the leakage of *access patterns*. We stored the plaintext instead of re-encrypting and storing it again on the server. In contrast, comparison schemes will increase server storage and *location*. As a result, searching for plaintext resulting in a continuous block is more efficient. Neither scheme reduces search *locality* since it involves touching index entries scattered in random places. Since our scheme only handles new entries after the last search, it achieves much better search performance. Consequently, there is a smaller *locality* for search results than alternative competitors.

### D. Decryption cost on the client’s side

**Fig 5:**
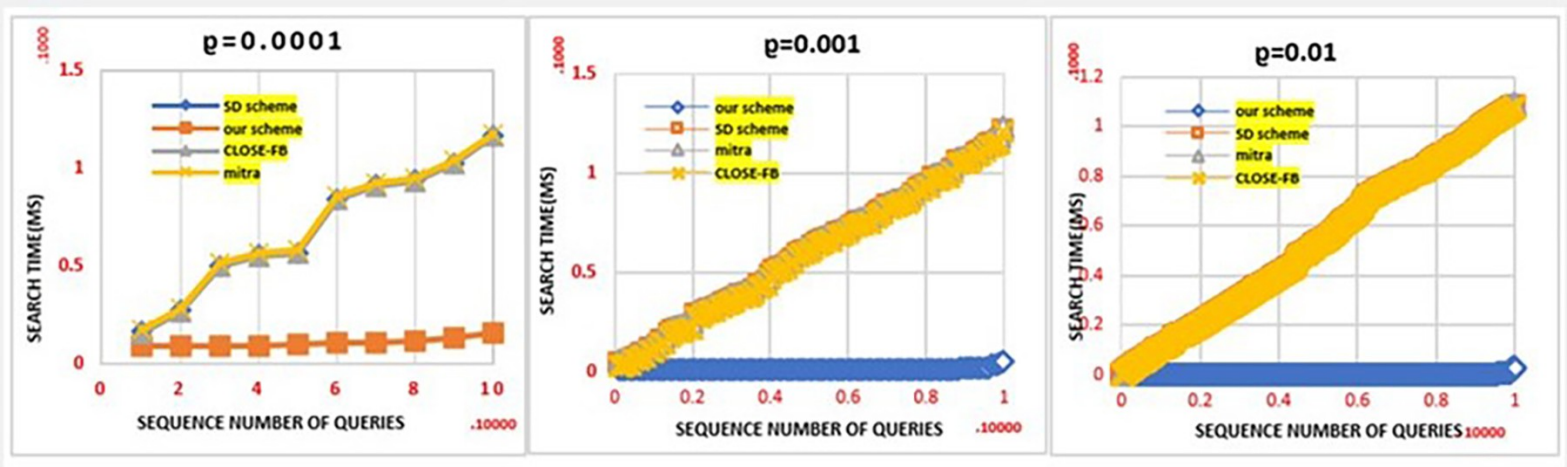
Dynamic decryption cost.

Due to the dynamic nature of our scheme, it will simulate the behavior of an entire system that operates dynamically, constantly updating and searching for specific keywords, as in the previous experiment. We designed a scheme that achieves backward privacy properties. During the update request, the keyword is preserved both upon addition or deletion on the server. When a search request is initiated, all past updates, including deletions and additions, are retrieved as results. Subsequently, the client filters out the deleted files, maintaining confidentiality regarding which files were deleted. This strategic approach prevents the server from creating links with subsequent update requests. To prevent the results of a search from being explicitly viewed, we retrieved the results from the server side in encrypted values that are not viewed explicitly during the search process, to ensure backward privacy. Our dynamic scheme offers the shortest decryption time for results retrieved from a server during a search, compared to other dynamic schemes, e.g., *Mitra*,*SD*_*d*_ and CLOSE−FB. Upon requesting a search for a specific keyword, the retrieved results are deleted from the server because these results consider access pattern leaks. Subsequently, the encrypted results are expressly preserved on the server. During subsequent searches, only the subsequent results are decrypted. According to these comparable schemes, each search query will decrypt all the results retrieved, even those retrieved previously, increasing client processing time. Thus, our scheme takes less time to decrypt encrypted values added after the last search on the client side. Fig 5 illustrates the situation.

In summary, The proposed scheme consistently excels in client storage efficiency, outperforming *Mitra* and *SD*_*d*_, while demonstrating lower query generation costs. In dynamic scenarios, it surpasses *Mitra* and CLOSE−FB in efficient search performance and ensures shorter client-side decryption times than Mitra, *SD*_*d*_, and CLOSE−FB. Overall, the proposed DSSE scheme exhibits superiority in storage and computational efficiency, dynamic search performance, and decryption speed over *Mitra* and *SD*_*d*_. Subsequent experiments indicate its superiority over CLOSE−FB in search performance and dynamic decryption cost, although specific figures for CLOSE−FB are not provided in the analysis.

**Remark:** It should be noted that the CLOSE−FB scheme was not added to Figs 2 and 3 because, when those experiments were performed, it was discovered that the client storage cost and query creation time of CLOSE−FB are similar to our scheme. However, when other experiments were performed, it was discovered that our scheme requires less time than CLOSE−FB, as shown in Figs 4 and 5.

## 6 Conclusion

Security features, such as forward and backward privacy, are crucial to preventing severe attacks on SSE. Using symmetric cryptographic primitives, we propose a dynamic SSE scheme that achieves forward privacy with a low client storage cost. Backward privacy is advanced by efficiently retrieving search results while concealing details about past updates. The solution should be implemented using a two-party model, in which the first party is the client. In contrast, the second party should act as the server, providing the client with extensive storage and efficient computation capabilities. The balanced distribution of responsibilities empowers both parties, optimizing the scheme for practical deployment in scenarios with dynamic data updates and the demand for efficient and secure search functionalities. The overall efficiency, including client storage, query generation costs, dynamic search performance, and decryption speed, is consistently demonstrated in comparison with *Mitra* and *SD*_*d*_. This thorough alignment between claims and experiment outcomes establishes a robust empirical basis, underscoring the proposed DSSE scheme’s superiority across multiple metrics. The experiments serve as validation, providing clear and direct connections between claimed advantages and observed outcomes, reinforcing the scheme’s efficacy. The alignment between specific experiment results and claims establishes a clear connection, providing empirical support for the proposed DSSE scheme’s advantages. We propose using an encrypted index at the server, to join keywords with the document identifiers that share keywords. A constant client storage cost for the DSSE framework is proposed in this paper, focusing on DSSE in practice. In order to evaluate the performance of our scheme, it was implemented alongside two previous schemes. The results of the experiments indicated that our scheme performs better than the best of the other existing schemes.

In future work, the pursuit of efficiency and performance improvements in dynamic searchable encryption schemes is crucial. This involves exploring computational methodologies to reduce processing time for search and database updates, optimizing overall scheme performance. Dynamic key management ensures ongoing security, while verification on the client and server sides becomes imperative, particularly in scenarios with unreliable servers. Scalability exploration is vital for efficient and secure large-scale search functionalities.
